# A systematic identification and analysis of scientists on Twitter

**DOI:** 10.1371/journal.pone.0175368

**Published:** 2017-04-11

**Authors:** Qing Ke, Yong-Yeol Ahn, Cassidy R. Sugimoto

**Affiliations:** School of Informatics and Computing, Indiana University, Bloomington, Indiana, United States of America; Administrative Headquarter, GERMANY

## Abstract

Metrics derived from Twitter and other social media—often referred to as altmetrics—are increasingly used to estimate the broader social impacts of scholarship. Such efforts, however, may produce highly misleading results, as the entities that participate in conversations about science on these platforms are largely unknown. For instance, if altmetric activities are generated mainly by scientists, does it really capture broader social impacts of science? Here we present a systematic approach to identifying and analyzing scientists on Twitter. Our method can identify scientists across many disciplines, without relying on external bibliographic data, and be easily adapted to identify other stakeholder groups in science. We investigate the demographics, sharing behaviors, and interconnectivity of the identified scientists. We find that Twitter has been employed by scholars across the disciplinary spectrum, with an over-representation of social and computer and information scientists; under-representation of mathematical, physical, and life scientists; and a better representation of women compared to scholarly publishing. Analysis of the sharing of URLs reveals a distinct imprint of scholarly sites, yet only a small fraction of shared URLs are science-related. We find an assortative mixing with respect to disciplines in the networks between scientists, suggesting the maintenance of disciplinary walls in social media. Our work contributes to the literature both methodologically and conceptually—we provide new methods for disambiguating and identifying particular actors on social media and describing the behaviors of scientists, thus providing foundational information for the construction and use of indicators on the basis of social media metrics.

## Introduction

Twitter and other social media have become important communication channels for the general public. It is thus not surprising that various stakeholder groups in science also participate on these platforms. Scientists, for instance, use Twitter for generating research ideas and disseminating and discussing scientific results [1–3]. Many biomedical practitioners use Twitter for engaging in continuing education (e.g., journal clubs on Twitter) and other community-based purposes [4]. Policy makers are active on Twitter, opening lines of discourse between scientists and those making policy on science [5].

Quantitative investigations of scholarly activities on social media—often called altmetrics—can now be done at scale, given the availability of APIs on several platforms, most notably Twitter [6]. Much of the extant literature has focused on the comparison between the amount of online attention and traditional citations collected by publications, showing low levels of correlation. Such low correlation has been used to argue that altmetrics provide alternative measures of impact, particularly the broader impact on the society [7], given that social media provide open platforms where people with diverse backgrounds can engage in direct conversations without any barriers. However, this argument has not been empirically grounded, impeding further understanding of the validity of altmetrics and the broader impact of articles.

A crucial step towards empirical validation of the broader impact claim of altmetrics is to identify scientists on Twitter, because altmetric activities are often assumed to be generated by “the public” rather than scientists, although it is not necessarily the case. To verify this, we need to be able to identify scientists and non-scientists. Although there have been some attempts, they suffer from a narrow disciplinary focus [8–10] and/or small scale [8, 10, 11]. Moreover, most studies use purposive sampling techniques, pre-selecting candidate scientists based on their success in other sources (e.g., highly cited in Web of Science), instead of organically finding scientists on the Twitter platform itself. Such reliance on bibliographic databases binds these studies to traditional citation indicators and thus introduces bias. For instance, this approach overlooks early-career scientists and favors certain disciplines.

Here we present the first large-scale and systematic study of scientists across many disciplines on Twitter. As our method does not rely on external bibliographic databases and is capable of identifying any user types that are captured in Twitter list, it can be adapted to identify other types of stakeholders, occupations, and entities. Our study serves as a basic building block to study scholarly communication on Twitter and the broader impact of altmetrics.

## Background

We classify current literature into two main categories, namely *product*- vs. *producer*-centric perspectives. The former examines the sharing of scholarly papers in social media and its impact, the latter focuses on who generates the attention.

**Product-centric perspective.** Priem and Costello formally defined Twitter citations as “direct or indirect links from a tweet to a peer-reviewed scholarly article online” and distinguished between first- and second-order citations based on whether there is an intermediate web page mentioning the article [12]. The accumulation of these links, they argued, would provide a new type of metric, coined as “altmetrics,” which could measure the broader impact beyond academia of diverse scholarly products [13].

Many studies argued that only a small portion of research papers are mentioned on Twitter [6, 14–19]. For instance, a systematic study covering 1.4 million papers indexed by both PubMed and Web of Science found that only 9.4% of them have mentions on Twitter [17], yet this is much higher than other social media metrics except Mendeley. The coverages vary across disciplines—medical and social sciences papers that may be more likely to appeal to a wider public are more likely to be covered on Twitter [19, 20]. Mixed results have been reported regarding the correlation between altmetrics and citations [17, 21–24]. A recent meta-analysis showed that the correlation is negligible (*r* = 0.003) [25]; however, there is dramatic differences across studies depending on disciplines, journals, and time window.

**Producer-centric perspective.** Survey-based studies examined how scholars present themselves on social media [26–30]. A large-scale survey with more than 3, 500 responses conducted by *Nature* in 2014 revealed that more than 80% were aware of Twitter, yet only 13% were regular users [29].

A handful of studies analyzed how Twitter is used by scientists. Priem and Costello examined 28 scholars to study how and why they share scholarly papers on Twitter [12]. An analysis of 672 emergency physicians concluded that many users do not connect to their colleagues while a small number of users are tightly interconnected [4]. Holmberg and Thelwall selected researchers in 10 disciplines and found clear disciplinary differences in Twitter usages, such as more retweets by biochemists and more sharing of links for economists [11].

Note that these studies first selected scientists outside of Twitter and then manually searched their Twitter profiles. Two limitations thus exist for these studies. First, the sample size is small due to the nature of manual searching [4, 8, 11, 12, 31]. Second, the samples are biased towards more well-known scientists. One notable exception is a study by Hadgu and Jäschke, who presented a supervised learning based approach to identifying researchers on Twitter, where the training set contains users who were related to some computer science conference handles [9, 32]. Although this study used a more systematic method, it still relied on the DBLP, an external bibliographic dataset for computer science, and is confined to a single discipline.

## Identifying scientists

### Scientist occupations

Defining science and scientists is a Herculean task and beyond the scope of this paper. We thus adopt a practical definition, turning to the 2010 Standard Occupational Classification (SOC) system (http://www.bls.gov/soc/) released by the Bureau of Labor Statistics, United States Department of Labor. We use SOC because not only it is a practical and authoritative guidance for the definition of scientists but also many official statistics (e.g., total employment of social scientists) are released according to this classification system. SOC is a hierarchical system that classifies workers into 23 major occupational groups, among which we are interested in two, namely (1) Computer and Mathematical Occupations (code 15-0000) and (2) Life, Physical, and Social Science Occupations (code 19-0000). Other groups, such as Management Occupations (code 11-0000) and Community and Social Service Occupations (code 21-0000), are not related to science occupations. From the two groups, we compile 28 scientist occupations (S1 Table). Although authoritative, the SOC does not always meet our intuitive classifications of scientists. For instance, “biologists” is not presented in the classification. We therefore consider another source—Wikipedia—to augment the set of scientist occupations. In particular, we add the occupations listed at http://en.wikipedia.org/wiki/Scientist#By_field.

We then compile a list of scientist titles from the two sources. This is done by combining titles from SOC, Wikipedia, and illustrative examples under each SOC occupation. We also add two general titles: “scientists” and “researchers.” For each title, we consider its singular form and the core disciplinary term. For instance, for the title “clinical psychologists,” we also consider “clinical psychologist,” “psychologists,” and “psychologist.” We assemble a set of 322 scientist titles using this method (S1 Data).

### List-based identification of scientists

**Fig 1:**
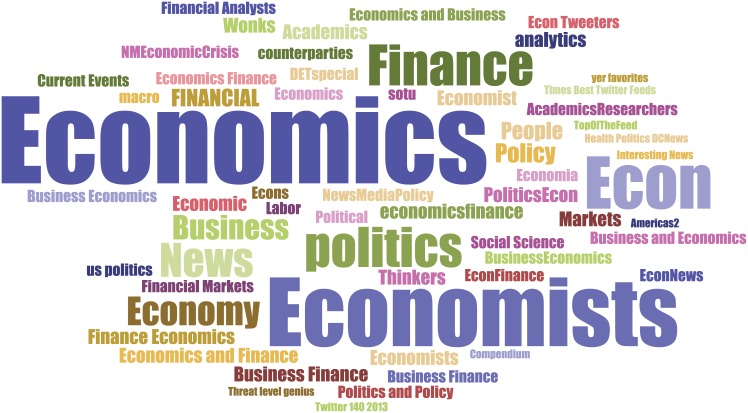
User identity recored from Twitter list names. We show the word cloud of Twitter lists containing @BetseyStevenson.

Our method of identifying scientists is inspired by a previous study that used Twitter *lists* to identify user expertise [33]. A Twitter *list* is a set of Twitter users that can be created by any Twitter user. The creator of a list needs to provide a name and optional description. Although the purpose of lists is to help users organize their subscriptions, the names and descriptions of lists can be leveraged to infer attributes of users in the lists. Imagine a user creating a list called “economist” and putting @BetseyStevenson in it; this signals that @BetseyStevenson may be an economist. If @BetseyStevenson is included in numerous lists all named “economist,” which means that many independent Twitter users classify her as an economist, it is highly likely that @BetseyStevenson is indeed an economist. This is illustrated in Fig 1 where the word cloud of the names of Twitter lists containing @BetseyStevenson is shown. We can see that “economist” is a top word frequently appeared in the titles, signaling the occupation of this user. In other words, we “crowdsource” the identity of each Twitter user.

In principle, we could use Twitter’s memberships API (https://dev.twitter.com/rest/reference/get/lists/memberships), for each user, to get all the lists containing this user, and then infer whether this user is a scientist by analyzing the names and descriptions of these lists. However, this method is highly infeasible, because (1) most users are not scientists, (2) the distribution of listed counts is right-skewed: Lady Gaga, for example, is listed more than 237*K* times (https://www.electoralhq.com/twitter-users/most-listed), and (3) Twitter API has rate limits. We instead employ a previously introduced list-based snowball sampling method [34] that starts from a given initial set of users and expands to discover more. We improve this approach by more systematically obtaining the job title lexicon, as described in the last section. Moreover, instead of choosing a few preselected users, we obtain a total of 8, 545 seed users by leveraging the results of a previous work that identified user attributes using Twitter lists [33] (S1 Text).

We use the snowball sampling (breadth-first search) on Twitter lists. We first identify seed users (S1 Text) and put them into a queue. For each public user in the queue, we get all the lists in which the user appears, using the Twitter memberships API. Then, for each public list in the subset resulting lists whose name contains at least one scientist title, we get its members using the Twitter members API (https://dev.twitter.com/rest/reference/get/lists/members) and put those who have not been visited into the queue. The two steps are repeated until the queue is empty, which completes the sampling process. Note that to remove many organizations and anonymous users as well as to speed up the sampling, we only consider users whose names contain spaces. We acknowledge that this may drop many users with non-English names or the ones who do not disclose their names in a standard way. Also note that this procedure is inherently blind towards those scientists who are not listed.

From the sampling procedure, we get 110, 708 users appearing in 4, 920 lists whose names contain scientist titles. To increase the precision of our method, the final dataset contains those users whose profile descriptions also contain scientist titles. A total number of 45, 867 users are found.

## Analyzing scientists

For each of the 45, 867 identified scientists, we obtain their followers, followings, and up to 3, 200 most recent statuses (tweets, retweets, and replies) using Twitter APIs. In total, we get 88, 412, 467 following pairs and 64, 449, 234 statuses. With this dataset, we ask the following questions:

- What are the demographics of identified scientists on Twitter, in terms of discipline and gender?
- What are the URLs scientists post in their tweets?
- How do scientists follow/retweet/mention each other on Twitter and who are the most “influential” scientists in these interactions?

These questions are necessary for the validation and appropriate utilization of altmetrics for research evaluation.

### Who are they?

We investigate the demographics of identified scientists in terms of discipline and gender.

#### Discipline

In contrast to previous analyses that either focused on a single discipline [8–10] and/or relied on a small number of accounts in a few disciplines [8, 10, 11], our systematic approach covers a wide range of disciplines, thus allowing us to investigate the representativeness of scientists in different disciplines. Moreover, identifying disciplines also allows us to analyze behavioral differences by disciplines and understand inter-disciplinary interactions between scientists.

To identify the discipline of each scientist, we leverage the compiled list of scientist titles. They are searched in profile descriptions and in assigned list names. Whereas profiles provide us information about how scientists perceive themselves, list names tell us how they are perceived by others. When searching, we begin with longer names and then move on to shorter ones. For instance, “I am an evolutionary biologist” will be matched with “evolutionary biologist” not with “biologist.” When multiple matches are found, each of them will be counted once. From profile descriptions, we obtain a total of 25, 798 (56.2%) users whose profile descriptions contain at least one scientist title, suggesting that a majority of “perceived” scientists identify themselves as scientists. S2 Table shows the number of users for each of the top 30 scientist titles extracted from profile descriptions. Psychologists are the most numerous, which may be rooted in two reasons. First, many types of psychology practitioners (e.g., counseling psychologists) are presented in the scientist titles. Second, many of them may not be resident in academia and serve as health care professionals. Thus, they may show this in their profiles to signal their profession. Clinical psychologists, for instance, are also highly represented. Other common type of scientists include physicists, computer scientists, and archaeologists.

When extracting titles from list names, there are 24, 635 (53.1%) users who are included in at least one list whose name contain scientist titles. S3 Table presents the number of users for each title. We observe some differences between the two rankings. Computer scientists, for instance, fail to make it into the top 10 based on list names, indicating that they are less often to be labeled by other users as “computer scientists” instead as other labels (e.g., “data scientists”). Sociologists, on the other hand, show the opposite trend.

Based on the titles extracted from profiles and list names, we now assign each user a final title or titles. We give more weight to titles from profiles by using profile information first when they are available. If this fails, we choose the title that appears the most times in the lists. With this procedure, we assign disciplines to 30, 793 (67.1%) users. Table 1 shows the number of users in the top 24 disciplines. These results again demonstrate that our method can discover scientists from diverse disciplines of sciences and social sciences.

**Table 1:** Number of users in most presented disciplines.

Discipline	Users	Discipline	Users
Historian	3586	Ecologist	775
Psychologist	3579	Anthropologist	698
Physicist	2737	Astronomer	675
Nutritionist	2510	Statistician	619
Political scientist	1441	Clinical psychologist	576
Computer scientist	1123	Linguist	526
Archaeologist	1100	Social scientist	438
Biologist	1075	Geographer	430
Economist	1044	Epidemiologist	403
Sociologist	1020	Mathematician	370
Neuroscientist	916	Geologist	359
Meteorologist	855	Evolutionary biologist	330

We investigate whether some disciplines are over- or under-represented in social media by comparing the results in Table 1 with the size of the science workforce. To do so, we use the total employment data from the latest (May 2014) National Occupational Employment Statistics (OES; http://www.bls.gov/oes/current/oes_nat.htm), which lists the size of workforce for each occupation. We aggregate the number of scientists onto the OES minor level (computer and information, mathematical, life, physical, and social scientists), and Table 2 shows the total number and the percentage of employment for each OES minor group as well as results from Twitter. These results suggest that social scientists and computer and information scientists are over-represented on Twitter, whereas mathematical, life, and physical scientists are under-represented. We should, however, note that (1) this is a rough estimation, as OES is solely for US but users in our sample may come from other countries, and (2) the results could also be biased due to our list-based sampling method. Therefore, further work is needed to check whether our results reflect an accurate representation on Twitter.

**Table 2:** Comparing number of scientists on Twitter and the size of the science workforce.

Title	Employment	Employment %	Twitter %	Ratio
Computer & Info.	24, 210	2.71%	3.62%	1.336
Mathematical	138, 540	15.48%	3.18%	0.205
Life	269, 660	30.13%	25.18%	0.836
Physical	274, 520	30.68%	19.66%	0.641
Social	187, 910	21.00%	48.37%	2.303

#### Gender

To identify gender, we first remove two common prefixes (“Dr.” and “Prof.”) and then search the first names in the 1990 US census database of frequently occurring first names (http://www.census.gov/topics/population/genealogy/data/1990_census/1990_census_namefiles.html), resulting in 11, 910 females and 18, 882 males. For the remaining unknown users, we detect their gender by using a facial feature detection service provided by Face++ (http://www.faceplusplus.com/detection_detect/). The input of this service is the URL of the image, which we use the profile image URL provided by Twitter, and one of its returned values is the gender associated with a confidence value. We only keep the gender results with confidence greater than 90. Combining the two methods, we are able to identify the gender for 71.9% (12, 732 females and 20, 232 males) of the sample. Of those identified, 38.6% were female and 61.4% were male, or the female to male ratio is 0.629.

We compare the female-male ratio of the sampled scientists on Twitter to the ones derived from two other samples, namely general Internet users and offline scientific authorships. A recent report from the Pew Research Center shows that 21% and 24% of female and male Internet users use Twitter (http://www.pewinternet.org/2015/01/09/demographics-of-key-social-networking-platforms-2/), leading to the female-male ratio 21%/24% = 0.875 (the ratio for Internet users is 0.99.). Regarding scientific authorships, the ratio ranges from 0.179 (Iran) to 0.754 (Poland), and is 0.428 for US [35]. Based on these, the gender ratio is less skewed for scientists on Twitter compared with scientific authorships in US, supporting the argument that Twitter provides more opportunities for diverse participation from women.

### What do they share?

**Fig 2:**
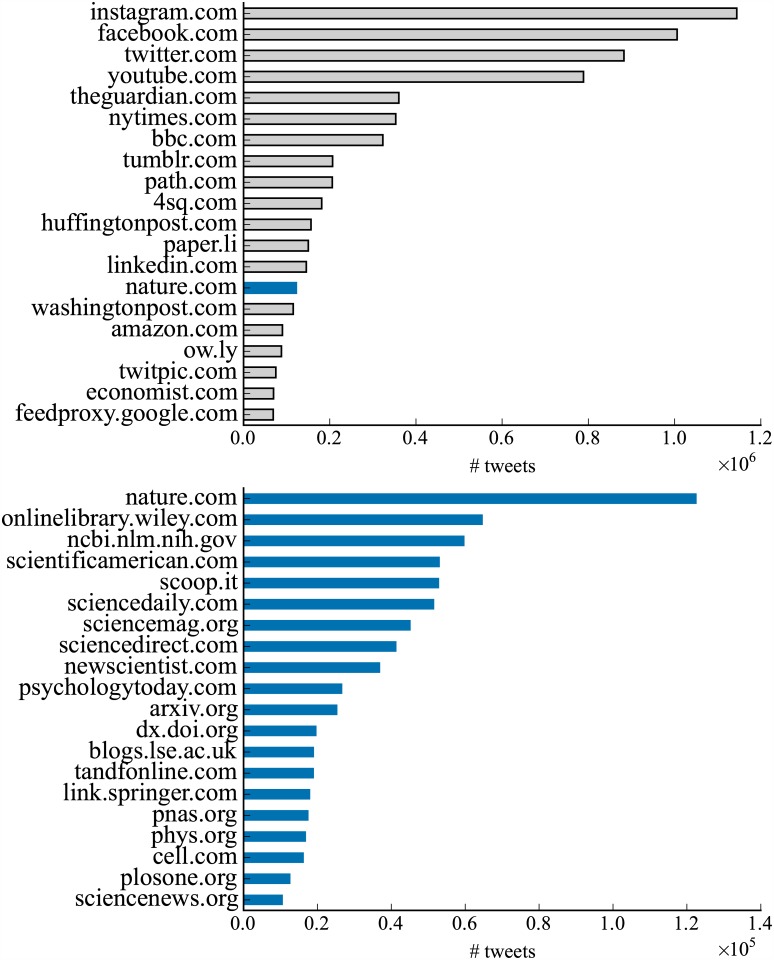
Top 20 domains. We extract URLs from tweets and retweets and then count the appearances of the domains. Top: overall. Bottom: scientific.

We study tweet contents posted by scientists. We specifically focus on URLs to understand sharing of scientific articles on Twitter. To do so, we extract URLs from tweets and retweets, ignoring replies. We only consider those generated from the retweet button as retweets and extract URLs from their original tweets. Noting that many top domains are shortened URLs (e.g., bit.ly), we expand them and extract domain names. Fig 2 (top) shows the top 20 domains and number of tweets mentioning them. We observe that many of them are to news websites, such as *The Guardian* and *The New York Times* and the domain for the Nature Publishing Group also ranks in the top. Fig 2 (bottom) displays the top scientific domains. Major academic publishers, such as Wiley (onlinelibrary.wiley.com), Elsevier (sciencedirect.com), Taylor &*amp*; Francis (tandfonline.com), and Springer (link.springer.com) appear in the top. Journals like *Science*, *PNAS*, and *PLoS ONE* also attract much attention on Twitter.

**Fig 3:**
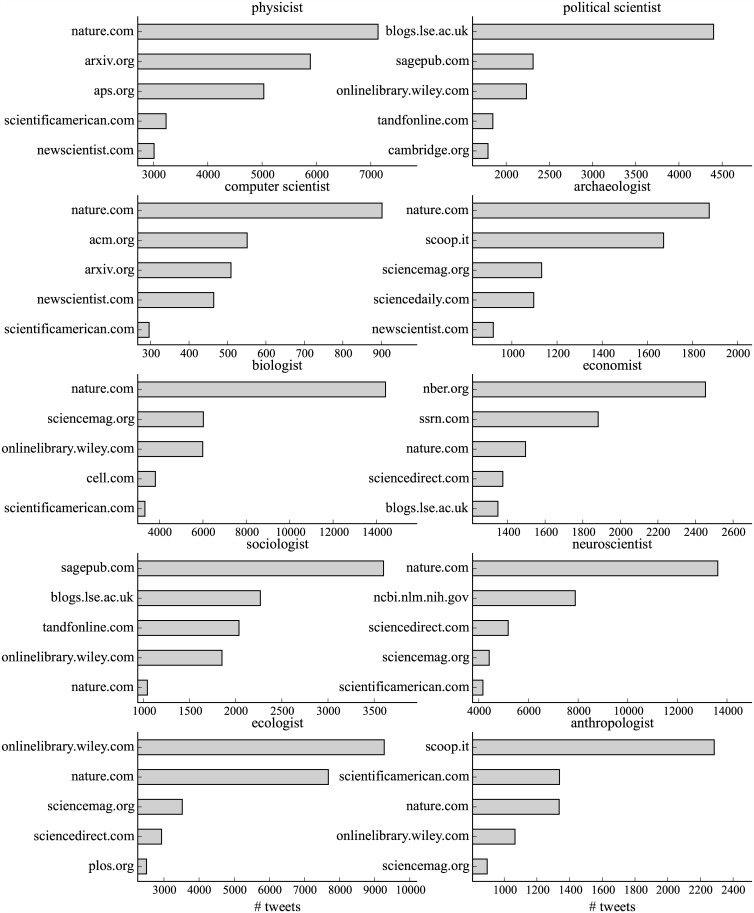
Top scientific domains by disciplines. We extract URLs from tweets and retweets and then count the appearances of the scientific domains in each of the 10 disciplines.

To understand disciplinary differences of the posted URLs, Fig 3 shows the top 5 scientific domains shared by scientists in each discipline. Although some domains such as nature.com are popular across disciplines, scientists are more likely to share content from their disciplines. For instance, arxiv.org, a pre-print server mainly for physics, and aps.org, the website for the American Physical Society, are the top domains for physicists. acm.org is popular among computer scientists. The blog for the London School of Economics and Political Science (LSE) (http://blogs.lse.ac.uk) is popular among political scientists, economists, and sociologists.

**Fig 4:**
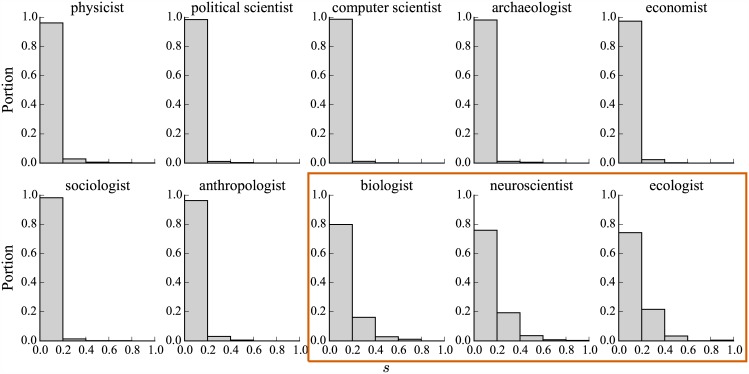
Distribution of fraction of scientific domains. For each scientist, *s* is the fraction of (re)tweets that contains URLs referring to scientific websites to the total number of (re)tweets that contains URLs. We show the histograms of *s* of scientists for each of the 10 disciplines.

To understand to what extent scientists share scientific URLs, we calculate for each user the fraction *s* of (re)tweets that contains URLs referring to scientific websites to the total number of (re)tweets that contains URLs. Fig 4 shows histograms of *s* by disciplines. Clearly, the fractions are small across all disciplines, while biological scientists—biologists, neuroscientists, and ecologists—post more tweets referring to scientific domains. For other types of scientists, the fraction is smaller than 0.2 for nearly all of them. This suggests that for most scientists on Twitter, sharing links to scientific domains is a minor activity.

### How do they connect to each other?

**Fig 5:**
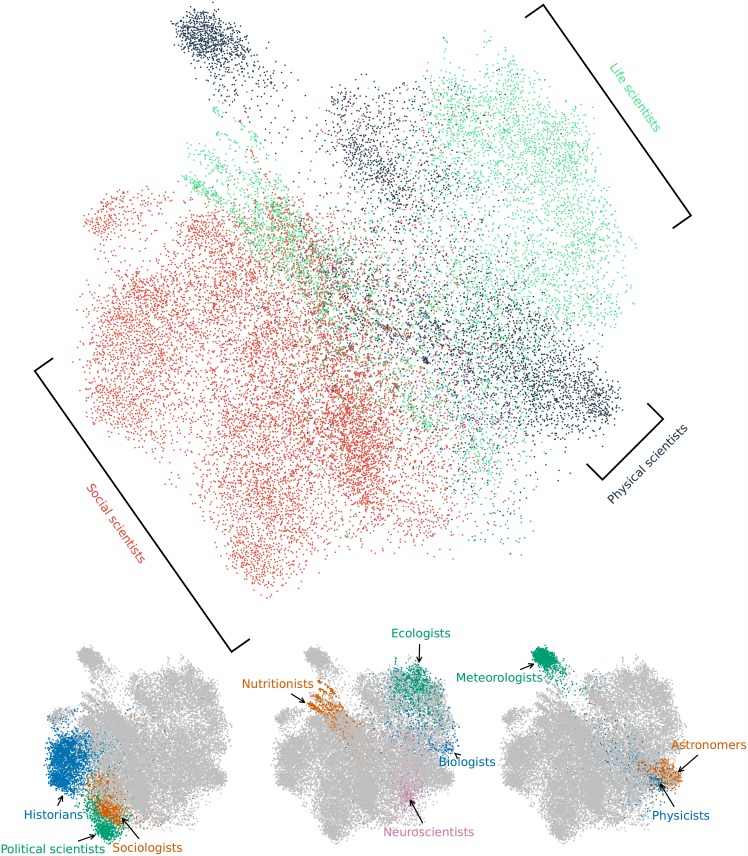
Follower network of scientists on Twitter. We use the ForceAtlas2 [36] algorithm to layout the largest connected component of the follower network with mutual following relations. We only show nodes with known disciplines.

We investigate how scientists connect with each other, by examining the follower, retweet, and mention networks between them. In the follower network, a directed and unweighted link from user *a* to *b* means that *a* follows *b*. In the retweet network, a directed link pointing from *a* to *b* is weighted, with the weight representing the number of times that *a* has retweeted *b*’s tweets. In the mention network, a link is also directed and weighted, and the weight indicates the number of times that *a* has mentioned *b* in *a*’s tweets. Table 3 reports summary statistics of the largest weakly connected components in the three networks. Fig 5 shows the follower network, where each node is a scientist and the color represents the extracted title.

**Table 3:** Summary statistics of scientist networks.

Network	Links	# nodes	# links
Follower	Who-follows-whom	39, 485	1, 234, 905
Retweet	Who-retweets-whom	30, 204	480, 479
Mention	Who-mentions-whom	26, 078	168, 232

#### Centralities

Given the three networks, we investigate the most influential—operationalized by network centralities—scientists on Twitter. S4 Table lists top scientists under the centrality of in-degree *d*_←_ or in-strength *s*_←_, PageRank *PR*, and *k*-core number *k* in the three networks. We observe that there are some overlaps between *d*_←_ (*s*_←_) and *PR* across the three networks and that top scientists in terms of *k*-core are different from those in terms of the other two measures. Regarding individual users, Neil deGrasse Tyson (@neiltyson), an astrophysicist, is ranked the first under degree and PageRank for all the three networks.

**Fig 6:**
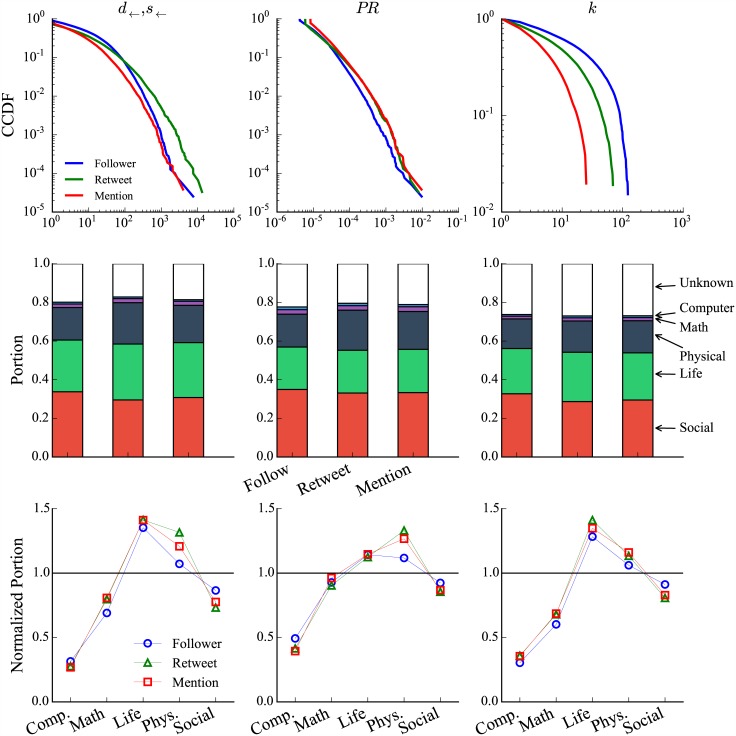
Distributions of centralities in the follower, retweet, and mention networks between scientists. Top: Distribution of in-degree *d*_←_ or in-strength *s*_←_, PageRank (*PR*), and *k*-core number *k* in the three networks. Middle: Portion of centralities occupied by scientists in each group. We calculate, for each centrality in each network, the sum of centrality values of users in each scientist group divided by the total centrality values. Bottom: Normalized portion of the three types of centralities occupied by scientists in each group in the three networks. Normalization is done by dividing the portion by number of scientists.

Going beyond top nodes, we show in Fig 6 (top) the distributions of centralities in the three networks. We observe that the distribution for *k*-core number is less heterogeneous than the other two centralities across the three networks, and the distributions of PageRank are similar for the three networks. The heterogeneity in centrality distributions raises the question of how attention is distributed among disciplines. We thus calculate, for each centrality in each network, the sum of centrality values of users in each OES minor group (mathematical, life, etc.) divided by the total values of the centrality. Fig 6 (middle) shows results. We can see that social and life scientists account for the largest part of centralities, followed by physical scientists. Mathematical and computer scientists only occupy a very small portion. Combining these results and Table 2, we further ask how this can be explained by the number of scientists in each minor group and whether scientists from some groups disproportionately account for the centralities. For each centrality in each network, we normalize the fraction of the centrality by the fraction of scientist in each group. Fig 6 (bottom) shows the normalized portion of centralities possessed by each OES minor group, where the result greater than one means that scientists in the group disproportionately have larger centralities, which is the case for life and physical scientists under all the three centralities in the three networks. Computer, mathematical, and social scientists exhibit the opposite pattern.

#### Assortativity

Assortativity quantifies the tendency that nodes with similar attributes are connected [37]. We observe from Fig 5 that scientists from the same discipline are positioned closely, signaling a positive assortativity with respect to discipline. This is indeed the case, as the assortativity coefficient is 0.548 (ignoring nodes with unknown discipline), indicating that the follower network is assortative by discipline—scientists tend to follow others from their own discipline group. However, this is not the case for gender (assortativity coefficient 0.054), implying that scientists follow others with the same gender not more often than expectation by pure chance. The retweet and mention networks are also assortative with respect to disciplines, with coefficient 0.492 and 0.537, but not to gender (coefficient 0.074 and 0.086).

## Discussion

Our work presents an improvement over earlier methods of identifying scientists on Twitter by selecting a wider array of disciplines and extending the sampling method beyond the paper-centric approach. Our method may serve as a useful step towards more extensive and sophisticated analyses of scientists on Twitter—it cannot be assumed that the population of scientists on Twitter is similar in composition and behavior to the population of scientists represented in traditional bibliometric databases. Therefore, sampling should be independent of these external data and metrics. Furthermore, in seeding with terms from the Standard Occupational Classification provided by the Bureau of Labor Statistics, we are able to classify both scholarly and practitioner scientific groups, thus widening the conceptualization of scientists on Twitter.

The triangulation of list- and bio-based classifications of scholars allows us to integrate two perspectives on identity: how scientists self-identified and how they were identified by the community. Our approach favors precision over recall; that is, we feel confident that those identified were scientists, but there is a much larger population of scientists who were not identified in this way.

Our disciplinary analyses suggest that Twitter is employed by scholars across the disciplinary spectrum—historians were widely represented, as were physicists, political scientists, computer scientists, biologists, economists, and sociologists. Practitioners were also highly represented—psychologists and nutritionists were in the top five in terms of disciplines with the highest number of identified members. However, a large percentage was also explicitly academic scholars: self-identified students and faculty members comprised 21.9% of the total population (S1 Text). Our analysis suggests that social scientists are overrepresented on Twitter, given their proportional representation in the scientific workforce, and that mathematicians are particularly underrepresented. Our findings resonate with some previous results [19], which looked at social media metric coverage of publications by field. They found higher Twitter density in the social and life sciences and lower density for mathematics and computer science. This provides some intuitive alignment: if a group is systematically underrepresented on the platform, we might expect a lower degree of activity around papers within that discipline.

Of those whose gender could be identified, 38.6% were female and 61.4% were male. This represents a more equal representation of women than seen in other statistics on the scientific workforce, such as number of publications [35], suggesting that Twitter scientists may be more gender-balanced than the population of publishing scientists.

As might be expected, scientists tweet in much the same way as the general population: Instagram, Facebook, YouTube are among the most tweeted domains, along with general news sites such as *The Guardian*, *New York Times*, and the *BBC*. However, scientists also have a distinct imprint of scholarly sites, such as generalists publications (i.e., *Nature* and *Science*) and reinforce the academic oligarchy of journal publishers [38]. The popular pre-print server, arXiv, also occupies a prominent spot among the top 20 cited domains. However, overall, tweets to these URLs identified as scientific only represented a small fraction of the overall tweets, suggesting that the content of scientists’ tweets is highly heterogeneous. This reinforces previous studies, which showed a strong blurring of boundaries between the personal and professional on Twitter, under a single Twitter handle [30].

We operationalized centralities in three ways: by followers, retweets, and mentions. Social and life scientists dominate these networks and mathematicians and computer scientists are relatively isolated. However, once these centralities are normalized by the size of the group, social scientists actually underperform, given their size. This is imperative information for the construction of indicators on the basis of these metrics. Just as it is standard bibliometric practice to normalize by field, so too should altmetric practices integrate normalization, given the uneven distribution of disciplines represented on these platforms.

Analysis of assortativity suggests that disciplinary communities prevail in the unfiltered realm of social media—scholars from the same disciplines tended to follow each other. This could suggest a negative result in terms of broader impact of social media metrics—if disciplinary walls are maintained in this space, it may not provide the unfettered access to scholarship that was promised. Furthermore, networks of communities reveal some isolation: e.g., although they represent a large proportion of the total users identified, historians are largely isolated in the Twitter network.

Our work has the following limitations. First, the reliance of Twitter lists leads to our method inherently blind towards those scientists who are not listed. Furthermore, the use of lists may skew towards the elite and high profile science communicators (e.g., Neil deGrasse Tyson). Second, in the sampling process, the exclusion of users whose names are without spaces biases the sample towards English-speaking users and causes many scientists not discovered. Third, the existence of private lists prohibits us to get the members there and affects further discovery of new users. Fourth, how list members were curated is largely unknown, and this might be done automatically and thus decrease the precision of identified scientists. Fifth, in the post-processing, the filtering of users whose profile descriptions do not contain scientist titles biases the sample towards self-disclosed scientists.

## Conclusion

In this work, we have developed a systematic method to discovering scientists who are recognized as scientists by other Twitter users through Twitter list and self-identify as scientists through their profile. We have studied the demographics of identified scientists in terms of discipline and gender, finding over-representation of social scientists, under-representation of mathematical and physical scientists, and a better representation of women compared to the statistics from scholarly publishing. We have analyzed the sharing behaviors of scientists, reporting that only a small portion of shared URLs are science-related. Finally, we find an assortative mixing with respect to disciplines in the follower, retweet, and mention networks between scientists.

Future work is needed to examine the use of machine learning methods [9] by leveraging information from retweet and mention networks to improve our identification method, to investigate the degree to which a more equal representation of women is due to age, status, or the representation of practitioners in our dataset, and to ascertain to what extent altmetric communities (i.e., follow, retweet, and mention networks) align with or differ from bibliometrically-derived communities (i.e., citation and collaboration networks).

## Supporting information

S1 Text (PDF)

S1 Fig Network of communities. (PDF)

S1 Table Scientist occupations from 2010 Standard Occupational Classification released by US Department of Labor. (PDF)

S2 Table Top scientist titles from profile descriptions. (PDF)

S3 Table Top scientist titles from Twitter list names. (PDF)

S4 Table Top scientists in the follower, retweet, and mention networks between scientists by in-degree *d*_←_ or in-strength *s*_←_, PageRank (*PR*), and *k*-core number. (PDF)

S5 Table Top users in each community. (PDF)

S1 Data List of scientist titles. (TXT)
