# Supplementary material for: A systematic identification and analysis of scientists on Twitter
Source: PLoS One. 2017 Apr 11;12(4):e0175368. doi: 10.1371/journal.pone.0175368 (PMC5388341; doi:10.1371/journal.pone.0175368)
Supplement: S1 Text — (PDF) [file pone.0175368.s001.pdf]

S1 Text

Getting initial seed users: One easy way to obtain the initial seed users is to use an established set of scientists, for instance, the top 100 science stars (http://news.sciencemag.org/scientific-community/2014/10/twitters-science-stars-sequel) compiled by *Science*. However, this may introduce bias towards more popular scientists and disciplines. Given our goal of identifying scientists at the scale of the entire Twitter platform, we instead take a more systematic approach by leveraging the results of a previous work that identified attributes of Twitter users [3]. The attributes of a user are the most frequently used words in the names and descriptions of the lists containing the user. These attributes are provided via the website http://twitter-app.mpi-sws.org/who-is-who that takes the screen name of a Twitter user as input and returns a word cloud for the given user with font sizes of words encoding the frequency of their appearance in list names and descriptions. Note that attributes are only available for those users who are included in at least 10 lists [3].

We first collect $285,760,507$ unique users by scanning a Twitter Gardenhose dataset, which contains about 10% of all public tweets from January 2013 to June 2014. The number of users is comparable to the number reported in a previous large-scale Twitter study [1], and the set of users covers any account that tweeted at least once and at least one of these tweets is included in Gardenhose during the period. We then filter out those users who were listed less than 8 times in our corpus, and query all the remaining users to the who-is-who website, finally obtaining attributes of $2,436,889$ users.

We then obtain seed users who are most likely to be scientists from the $2.4M$ users. As the seeds will be used for expansion, we prefer precision to recall. We thus adopt stringent criteria to filter out non-seed users. Specifically, we disregard the least important attributes of each user and then keep those users whose attributes contain the attribute "science" and at least one scientist title compiled before. The obtained initial set has $8,545$ users, and we use them as initial seeds for snowball sampling.

Academic rank: It is also interesting to investigate academics and to understand how scientists with different academic ranks (PhD student, postdoc, and professor) are represented on Twitter. We extract this information by searching for the following

keywords in profile descriptions:

- student: *phd student, phd candidate, graduate student, grad student, doctoral student*;
- postdoc: *postdoc, post-doc, postdoctoral*;
- professor: *assistant professor, assistant prof, asst prof, associate professor, associate prof, assoc prof, professor, prof, faculty.*

When more than one category are found, we choose the one that appears first. We identify $3,705$ students, $1,030$ postdocs, and $5,326$ professors. This indicates that many professors disclose their professional information on Twitter.

Community structure: We understand the follower network from the mesoscopic view—community structure. Analysis of communities helps us understand how scientists' following activities are organized and what the scholarly communities online are. These results will further advance our understanding of the role of disciplines in the interactions between scientific communities and of the comparisons with offline collaboration or citations networks.

To identify communities in the follower network, we employed the Infomap algorithm [2] and identified 343 communities with more than 10 nodes. Fig S1 shows the network between the top 15 communities. The number of links is set as the minimum value that keeps the network connected. To understand what these communities are, we count the appearance of individual words (excluding stop-words (a, and, of, the, in, at, to, i, for, your, on, are, my, own, with)) in the profile descriptions of users in each community. We use the top five most appeared words to label each community, as showed in Fig S1. We can see that scientists seem to organize based on disciplines. They follow other scientists in their own scientific communities. The two communities that are composed with ecologists and biologists are tightly connected with each other. This is also the case for (1) astronomers and physicists, and (2) political scientists, economist, and sociologist. In Table S5, we report the top scientists in each community based on their PageRank.

References

1. M. Gabielkov, A. Rao, and A. Legout. Studying social networks at scale: macroscopic anatomy of the twitter social graph. In *Proc. of the 2014 ACM International Conference on Measurement and Modeling of Computer Systems*, pages 277–288, 2014.

2. M. Rosvall and C. T. Bergstrom. Maps of random walks on complex networks reveal community structure. *PNAS*, 105:1118–1123, 2008.

3. N. K. Sharma, S. Ghosh, F. Benevenuto, N. Ganguly, and K. Gummadi. Inferring who-is-who in the twitter social network. In *Proc. of the 2012 ACM Workshop on Online Social Networks*, pages 55–60, 2012.
