# Supplementary figures and images for: A systematic identification and analysis of scientists on Twitter
Source: PLoS One. 2017 Apr 11;12(4):e0175368. doi: 10.1371/journal.pone.0175368 (PMC5388341; doi:10.1371/journal.pone.0175368)

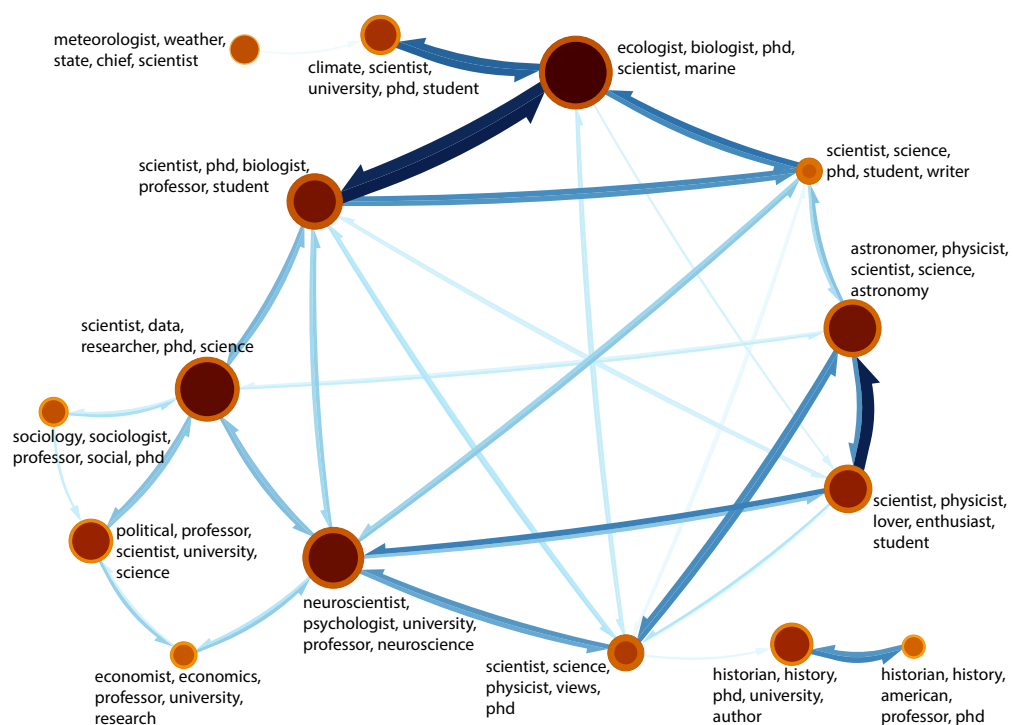

Fig S1. Network of communities.

Supplement: S1 Fig — (PDF) [file pone.0175368.s002.pdf]
