# Supplementary material for: A systematic identification and analysis of scientists on Twitter
Source: PLoS One. 2017 Apr 11;12(4):e0175368. doi: 10.1371/journal.pone.0175368 (PMC5388341; doi:10.1371/journal.pone.0175368)
Supplement: S1 Table — (PDF) [file pone.0175368.s003.pdf]

Table S1. Scientist occupations from 2010 Standard Occupational Classification released by US Department of Labor.

15-1100 Computer Occupations	
15-1111	Computer and Information Research Scientists
15-2000 Mathematical Science Occupations	
15-2021	Mathematicians
15-2041	Statisticians
19-1000 Life Scientists	
19-1011	Animal Scientists
19-1012	Food Scientists
19-1013	Soil and Plant Scientists
19-1021	Biochemists and Biophysicists
19-1022	Microbiologists
19-1023	Zoologists and Wildlife Biologists
19-1031	Conservation Scientists
19-1041	Epidemiologists
19-1042	Medical Scientists
19-2000 Physical Scientists	
19-2011	Astronomers
19-2012	Physicists
19-2021	Atmospheric and Space Scientists
19-2031	Chemists
19-2032	Materials Scientists
19-2041	Environmental Scientists
19-2042	Geoscientists
19-2043	Hydrologists
19-3000 Social Scientists and Related Workers	
19-3011	Economists
19-3031	Clinical, Counseling, and School Psychologists
19-3032	Industrial-Organizational Psychologists
19-3041	Sociologists
19-3091	Anthropologists and Archeologists
19-3092	Geographers
19-3093	Historians
19-3094	Political Scientists
