# Supplementary material for: A systematic identification and analysis of scientists on Twitter
Source: PLoS One. 2017 Apr 11;12(4):e0175368. doi: 10.1371/journal.pone.0175368 (PMC5388341; doi:10.1371/journal.pone.0175368)
Supplement: S2 Table — (PDF) [file pone.0175368.s004.pdf]

Table S2. Top scientist titles from profile descriptions.

Discipline	Users	Discipline	Users
Psychologist	3379	Sociologist	538
Historian	2826	Astronomer	463
Physicist	2561	Social scientist	343
Nutritionist	2468	Mathematician	333
Computer scientist	1089	Linguist	320
Archaeologist	919	Geographer	319
Political scientist	891	Epidemiologist	294
Biologist	866	Genealogist	254
Meteorologist	818	Geologist	253
Ecologist	698	Chemist	242
Neuroscientist	665	Astrophysicist	236
Economist	661	Microbiologist	214
Statistician	599	Environmental scientist	208
Clinical psychologist	576	Evolutionary biologist	194
Anthropologist	546	Pathologist	177
