# Supplementary material for: A systematic identification and analysis of scientists on Twitter
Source: PLoS One. 2017 Apr 11;12(4):e0175368. doi: 10.1371/journal.pone.0175368 (PMC5388341; doi:10.1371/journal.pone.0175368)
Supplement: S3 Table — (PDF) [file pone.0175368.s005.pdf]

Table S3. Top scientist titles from Twitter list names.

Discipline	Users	Discipline	Users
Psychologist	4663	Epidemiologist	387
Historian	3371	Geographer	357
Physicist	2859	Geologist	344
Nutritionist	2510	Evolutionary biologist	336
Archaeologist	1183	Genealogist	298
Sociologist	996	Social scientist	281
Economist	955	Ecologist	193
Biologist	889	Geoscientist	188
Meteorologist	824	Social psychologist	181
Astronomer	768	Pathologist	172
Political scientist	756	Astrophysicist	159
Anthropologist	629	Mathematician	148
Statistician	594	Microbiologist	143
Neuroscientist	571	Entomologist	126
Linguist	476	Chemist	121
