# Supplementary material for: A systematic identification and analysis of scientists on Twitter
Source: PLoS One. 2017 Apr 11;12(4):e0175368. doi: 10.1371/journal.pone.0175368 (PMC5388341; doi:10.1371/journal.pone.0175368)
Supplement: S4 Table — (PDF) [file pone.0175368.s006.pdf]

Table S4. Top scientists in the follower, retweet, and mention networks between scientists by in-degree $d_{\leftarrow}$ or in-strength $s_{\leftarrow}$, PageRank (PR), and k-core number.

$d_{\leftarrow}$	neiltyson, RichardDawkins, sapinker, phylogenomics, donttrythis
PR	neiltyson, RichardDawkins, sapinker, SamHarrisOrg, paulbloomatyale
k	randal_olson, Write4Research, zacharyapte, abcsoka, ballenamar
$s_{\leftarrow}$	neiltyson, AstroKatie, elakdawalla, phylogenomics, WhySharksMatter
PR	neiltyson, AstroKatie, conradhackett, RichardDawkins, elakdawalla
k	phylogenomics, surt_lab, duffy_ma, SciBry, ethanwhite
$s_{\leftarrow}$	neiltyson, phylogenomics, RichardDawkins, WhySharksMatter, AtheneDonald
PR	neiltyson, RichardDawkins, sapinker, elakdawalla, phylogenomics
k	raulpacheco, CMBuddle, mocost, imascientist, davenuss79
