# Supplementary material for: A systematic identification and analysis of scientists on Twitter
Source: PLoS One. 2017 Apr 11;12(4):e0175368. doi: 10.1371/journal.pone.0175368 (PMC5388341; doi:10.1371/journal.pone.0175368)
Supplement: S5 Table — (PDF) [file pone.0175368.s007.pdf]

Table S5. Top users in each community.

	Top users
1	JacquelynGill, GlobalEcoGuy, SylviaEarle
2	zephoria, EdwardTufte, hmason
3	paulbloomatyale, danariely, deevybee
4	elakdawalla, seanmcarroll, AstroKatie
5	phylogenomics, EricTopol, JCVenter
6	neiltyson, RichardDawkins, sapinker
7	kinggary, FukuyamaFrancis, BrendanNyhan
8	holland_tom, JamesThorne2, EZuelow
9	MichaelEMann, KHayhoe, ClimateOfGavin
10	jimalkhalili, DrAliceRoberts, RogerHighfield
11	JimCantore, DrShepherd2013, reedtimmerTVN
12	conradhackett, alondra, lisawade
13	TimHarford, R_Thaler, CassSunstein
14	deborahblum, kejames, KateClancy
15	wcronon, TomSugrue, samueljredman
